# Tumor purity–associated genes influence hepatocellular carcinoma prognosis and tumor microenvironment

**DOI:** 10.3389/fonc.2023.1197898

**Published:** 2023-06-26

**Authors:** Yan Zhao, Xu Xu, Yue Wang, Lin D. Wu, Rui L. Luo, Ren P. Xia

**Affiliations:** ^1^ Department of Organ Transplantation, Kunming Medical University First Affiliated Hospital, Kunming, China; ^2^ Department of Urology, The First Affiliated Hospital of Chengdu Medical College, Chengdu, China

**Keywords:** hepatocellular carcinoma, tumor microenvironment, tumor purity, prognostic gene, bioinformation analysis

## Abstract

**Introduction:**

Tumor purity takes on critical significance to the progression of solid tumors. The aim of this study was at exploring potential prognostic genes correlated with tumor purity in hepatocellular carcinoma (HCC) by bioinformatics analysis.

**Methods:**

The ESTIMATE algorithm was applied for determining the tumor purity of HCC samples from The Cancer Genome Atlas (TCGA). The tumor purity–associated genes with differential expression (DEGs) were identified based on overlap analysis, weighted gene co-expression network analysis (WGCNA), and differential expression analysis. The prognostic genes were identified in terms of the prognostic model construction based on the Kaplan–Meier (K–M) survival analysis and Least Absolute Shrinkage and Selection Operator (LASSO) regression analyses. The expression of the above-described genes was further validated by the GSE105130 dataset from the Gene Expression Omnibus (GEO) database. We also characterized the clinical and immunophenotypes of prognostic genes. Gene set enrichment analysis (GSEA) was carried out for exploring the biological signaling pathway.

**Results:**

A total of 26 tumor purity–associated DEGs were identified, which were involved in biological processes such as immune/inflammatory responses and fatty acid elongation. Ultimately, we identified ADCK3, HK3, and PPT1 as the prognostic genes for HCC. Moreover, HCC patients exhibiting higher ADCK3 expression and lower HK3 and PPT1 expressions had a better prognosis. Furthermore, high HK3 and PPT1 expressions and low ADCK3 expression resulted in high tumor purity, high immune score, high stromal score, and high ESTIMATE score. GSEA showed that the abovementioned prognostic genes showed a significant correlation with immune-inflammatory response, tumor growth, and fatty acid production/degradation.

**Discussion:**

In conclusion, this study identified novel predictive biomarkers (ADCK3, HK3, and PPT1) and studied the underlying molecular mechanisms of HCC pathology initially.

## 1 Introduction

Hepatocellular carcinoma (HCC) has been confirmed as the most frequently reported primary liver cancer, that is, the sixth most common cancer worldwide (1, 2). It is estimated that about 80% of liver cancer cases are HCC (3–5). HCC was a polygenic disease caused by the interaction of a variety of tumor-promoting and tumor-suppressing genes with the tumor microenvironment (TME). However, its molecular mechanism remains unclear (6, 7). HCC is subjected to poor prognosis, and the major reason for this drawback arises from its late presentation and limited therapeutic options (8). Currently, surgery and chemotherapy are the mainstays of treatment for HCC, whereas the incidence of HCC continues to rise and is increasing more rapidly than any other cancer in men and women. HCC ranks among the top 10 cancers for morbidity and mortality, as reported by the systematic analysis of the Global Burden of Disease Study (GBD) (9). Since most HCC patients are diagnosed late, they too weak to resist the risk of surgery. Accordingly, it is urgent to find new diagnostic and prognostic markers for HCC, which may help in early diagnosis and guide treatment decisions for improving patient survival and quality of life. Tumor purity has been defined as the proportion of tumor cells in the TME (10), TME is a cell population composed of a wide variety of cells (e.g., stromal cells, fibroblasts, endothelial cells, and immune cells), and this cell population takes on critical significance to tumors’ occurrence and development (11). Moreover, cellular and molecular components in the TME may exert certain effects on treatment outcomes (12). Existing research has suggested that tumor purity may affect co-expression networks, cluster-based classification of tumor sub-types or molecular classification, and identification of differentially expressed genes (13). However, tumor purity–associated markers in HCC have been rarely investigated.

In clinical practice, generally obtained solid tumors constantly comprise multiple clonal populations of cancer cells and adjacent normal tissue, stroma, and infiltrating immune cells, with a high degree of heterogeneity (14). This heterogeneous structure is capable of complicating or biasing the analysis of various genomic data. ESTIMATE has been adopted for the assessment of tumor purity in existing studies using gene expression signatures to determine the proportion of stromal and immune cells in a tumor sample (15). Furthermore, the algorithm has been reported as a robust tumor purity prediction algorithm. A total of 10 tumor purity prognosis-associated genes were extracted from this study for the investigation of tumor purity (16).

However, there is no immune-associated prognostic analysis of tumor purity in liver cancer. This study aims to investigate the correlation and mechanism between tumor purity and clinical prognosis in HCC, which devotes to better clarify prognostic prediction and therapeutic strategies. The analytical method in this study draws much inspiration from existing research (17).

## 2 Materials and methods

### 2.1 Data source

The Cancer Genome Atlas (TCGA) database provided RNA-sequencing data (i.e., 50 normal samples and 374 HCC samples). A total of 342 HCC samples were obtained after excluding samples from patients with incomplete clinical and survival information (**Supplementary Table S1**). The GSE105130 dataset originated from the GEO database (18) (https://www.ncbi.nlm.nih.gov/geo/query/acc.cgi?acc=GSE105130), containing transcriptomic data from paired 25 HCC patients with tumors and adjacent non-tumors. Moreover, the Gene Expression Omnibus (GEO; https://www.ncbi.nlm.nih.gov/geo/) database provided GSE36376 dataset with 240 HCC patients and 193 adjacent non-tumor samples. Data were downloaded from the publicly available database; hence, it was not applicable for additional ethical approval.

### 2.2 Differential expression analysis

In accordance with the RNA-sequencing data of 50 normal and 374 HCC samples in the TCGA database, differential expression analysis was carried out with the use of the R package limma. In accordance with the HCC versus normal comparison method, genes satisfying |log2fold change (FC)| > 0.5 and *P*< 0.05 were considered differentially expressed genes (DEGs). A volcano plot and a heat map were employed for demonstrating the distribution and expression patterns of DEGs.

### 2.3 Tumor purity and immune landscape

RNA-sequencing data of 374 HCC samples from the TCGA database were extracted as the basis for this step of the analysis. The immune score, stroma score, and ESTIMATE score of all TCGA-HCC samples were assessed using the ESTIMATE algorithm (R package ESTIMATE) (16). Equation 1 was adopted to estimate the respective HCC patient’s tumor purity:


(1) Tumor purity=cos(0.6049872018+0.0001467884×ESTIMATE Score)

The single-sample gene set enrichment analysis (ssGSEA) was further used to assess the abundance of 24 immune cell species in the TCGA-HCC (*n* = 374) cohort, and the result was visualized into a stacked plot.

### 2.4 Weighted Gene Co-expression Network Analysis

Weighted Gene Co-expression Network Analysis (WGCNA) refers to an analytical method for the analysis of gene expression patterns of multiple samples, allowing clustering of genes with similar expression patterns and analysis of correlations between modules and specific traits or phenotypes (19). For filtering 374 HCC samples, goodSamplesGenes function in R package WGCNA was employed for the first time, and the samples with TURE results were introduced into subsequent analyses. Tumor purity and immune infiltrating cell abundance were the specific traits of interest, and they are listed in **Supplementary Table S2**, with the aim of identifying tumor purity–associated genes.

Afterward, a scale-free co-expression network was built based on a soft-threshold parameter β (β was a soft-threshold parameter that could enhance strong correlations between genes and penalize weak correlations) using the adjacency matrix (20).

Subsequently, in accordance with the standard of hybrid dynamic tree cutting algorithm, the minimum number of genes in the respective gene module was set to 50, and MEDissThres was set to 0.2 to merge similar modules analyzed using the dynamic cut tree algorithm.

To identify the gene modules that are immune-associated to tumor purity in HCC, the tumor purity in HCC was defined as the trait was, and the relationship of the respective module and tumor purity was analyzed. The module with the maximum absolute value of the immune correlation coefficient with tumor purity was defined as a critical module for subsequent analysis, with *p*< 0.05 as the threshold with statistical significance, and genes in the vital module were considered hub genes.

### 2.5 Identification and enrichment analyses of tumor purity–associated genes with differential expression

Common genes between DEGs and hub genes were obtained through the overlap analysis, which were defined as tumor purity–associated DEGs. To explore whether there is an interaction between the tumor purity–associated DEGs, we used the STRING (https://string-db.org) website to construct a PPI network for them with a confidence level of 0.4. Subsequently, functional enrichment analyses were performed on tumor purity–associated DEGs by the R package clusterProfiler for Gene Ontology (GO) and Kyoto Encyclopedia of Genes and Genomes (KEGG), and *p*< 0.05 was regarded as notably enriched.

### 2.6 Survival analysis

For the assessment of whether tumor purity–associated DEGs were correlated with patient survival, we performed a K–M analysis by R-package survival. In brief, patients were divided into high- and low-expression groups based on whether the expression of the tumor purity–associated DEGs was greater than the median expression level of each tumor purity–associated DEG in the TCGA-HCC cohort. The K–M curve of each expression group of each tumor purity–associated DEGs was plotted and compared. The survival difference between high- and low-expression groups with *P*< 0.05 was considered as a gene notably correlated with OS in HCC patients.

### 2.7 Least Absolute Shrinkage and Selection Operator algorithm

Based on the 342 TCGA-HCC cases with complete survival and clinical information, gene notably correlated with OS obtained in the previous step were introduced into Least Absolute Shrinkage and Selection Operator (LASSO) algorithm with the parameters “famil” to “binomial” and “type. measure” to “class,” to select strong correlation features and obtain prognostic genes when the cross-validation error was the lowest.

To evaluate the prognostic value of the risk model, the risk score of each HCC sample was firstly calculated according to the expressions of prognostic genes and the risk coefficient obtained by LASSO with the formula: risk score. Then, the HCC samples were divided into high- and low-risk groups based on the optimal threshold of the risk score calculated by the surv cutpoint function of the R package survminer. Subsequently, a K–M survival difference analysis was employed on the high- and low-risk groups.

### 2.8 Establishment of a nomogram

The prognostic genes were further enrolled in developing a nomogram using rms and survival packages to predict the survival rate of patients with HCC, and riskRegression and survival packages were applied to plot calibration curve and quantified data of each risk group.

### 2.9 Clinical and immunological phenotypes of prognostic genes

First, clinical characteristics, covering T-phase (T1–T4), N-phase (N0 and N1), M-phase (M0 and M1), and PHASE (phase I–phase IV) were extracted from the 342 HCC samples. Then, the expression of each model gene was compared between subgroups of each clinical characteristic. To analyze the correlation between the expression of prognostic genes and the clinical characteristics of HCC, we compared the differences in the expression of three prognostic genes according to the clinical characteristics of different groups.

Moreover, based on the median expression value of the respective model gene, the HCC samples were separated into high- and low-expression groups of the respective model gene. Subsequently, CD8.T.cells, stromal scores, ESTIMATE scores, and tumor purity were compared between high and low expression groups of each model gene.

Moreover, the Pearson correlation of each model gene with the immune score, stromal score, ESTIMATE score, CD8.T.cells, and tumor purity was determined.

### 2.10 Single-gene gene set enrichment analysis

To investigate the relevant pathways and biological functions of the prognostic genes, the prognostic genes served as target genes, and the correlation coefficients between all genes in HCC samples and the expression levels of target genes were considered the sorting standards in terms of GSEA, which was performed by clusterProfiler with the selection standards of adjusted *p*< 0.05.

### 2.11 Validation of expression levels of prognostic genes

To further verify the expression levels of the prognostic genes, the expression of each model gene was compared between normal and HCC samples in TCGA cohort and GSE105130 dataset respectively. Furthermore, human hepatic stellate cell line LX-2, as well as three human HCC cell lines HepG2, SK-HeP-1, and Huh-1 were purchased from Procell Life Science and Technology Co., Ltd. (Wuhan, China). Total RNA from the above four cell lines (logarithmic phage) was segmented *via* the TRIzol Reagent based on the producer’s indications (Ambion, TX, USA). For the next processes, the inverse transcription of RNA into cDNA was done *via* the SweScript-First-strand-cDNA-synthesis-kit (Servicebio, Wuhan, China) and qPCR was completed through the 2× SYBR Green qPCR Master Mix depending on the manuals’ indications (Servicebio, Wuhan, China). The detailed information of primers synthesized by Beijing Tsingke Biotech Co., Ltd. (Beijing, China) is listed in **Table 1**. The relative expression of the respective prognostic gene was uniformized by GAPDH. The student’s *t*-test was carried out to compute the *P*-values between two groups. The *P*-value< 0.05 (two-tailed) was delimited as statistically significant.

**Table 1:** The detailed information of primers for qPCR.

Primer name	Primer sequence
PPT1 For	GAGGACGTGGAGAACAGCTT
PPT Rev	GGCATCGAGGGAGTCCAAAA
HK3 For	TGAGGTTGGGCTAGTTGTAGACAC
HK3 Rev	TGAGCACCAGGATTCAGGGA
ADCK3 For	CAGCCAGGAGATTCGGAACG
ADCK3 Rev	TATGGATTTCGCCCGCACA
GAPDH For	CGAAGGTGGAGTCAACGGATTT
GAPDH Rev	ATGGGTGGAATCATATTGGAAC

### 2.12 Statistical analysis

All statistical analyses were carried out using R software (version 3.5.2) and the relevant software packages. The specific statistical methods were stated in the relevant sections. Without special remarks, *P*< 0.05 was considered with statistical significance.

## 3 Results

### 3.1 Identification of the hepatocellular carcinoma–associated genes with differential expression

**Figure 1:**
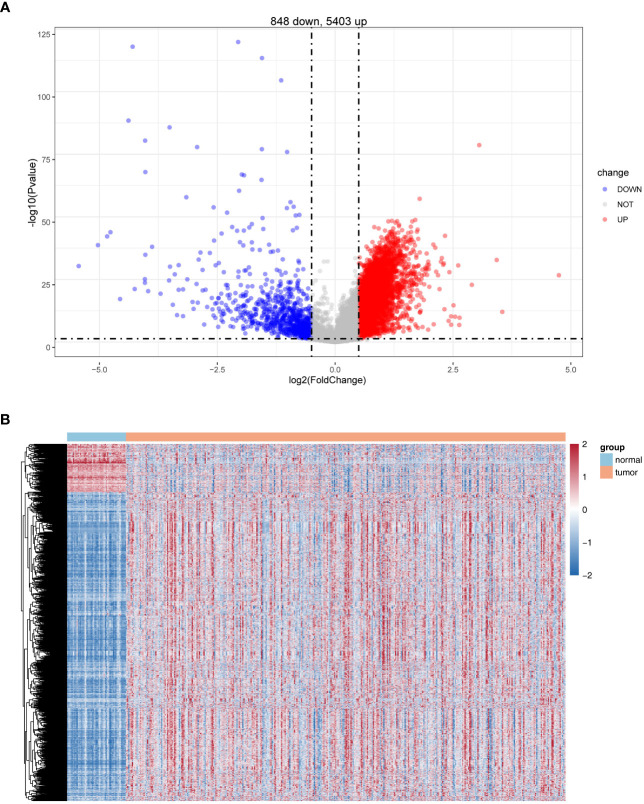
Identification of differentially expressed genes (DEGs). **(A)** Volcano plot of DEG (HCC *vs.* normal). **(B)** Heatmaps of DEGs (HCC *vs.* normal).

Differential expression analysis was conducted for 50 normal and 374 HCC samples in the TCGA database using the R package limma. Based on |log2 FC| > 0.5 and *P*< 0.05, a total of 6251 DEGs were identified, of which 848 were upregulated and 5,403 were downregulated in HCC samples in comparison with normal samples (**Figure 1**; **Supplementary Table S3**).

### 3.2 Analysis of tumor purity and immune infiltrating cell co-expression network

**Figure 2:**
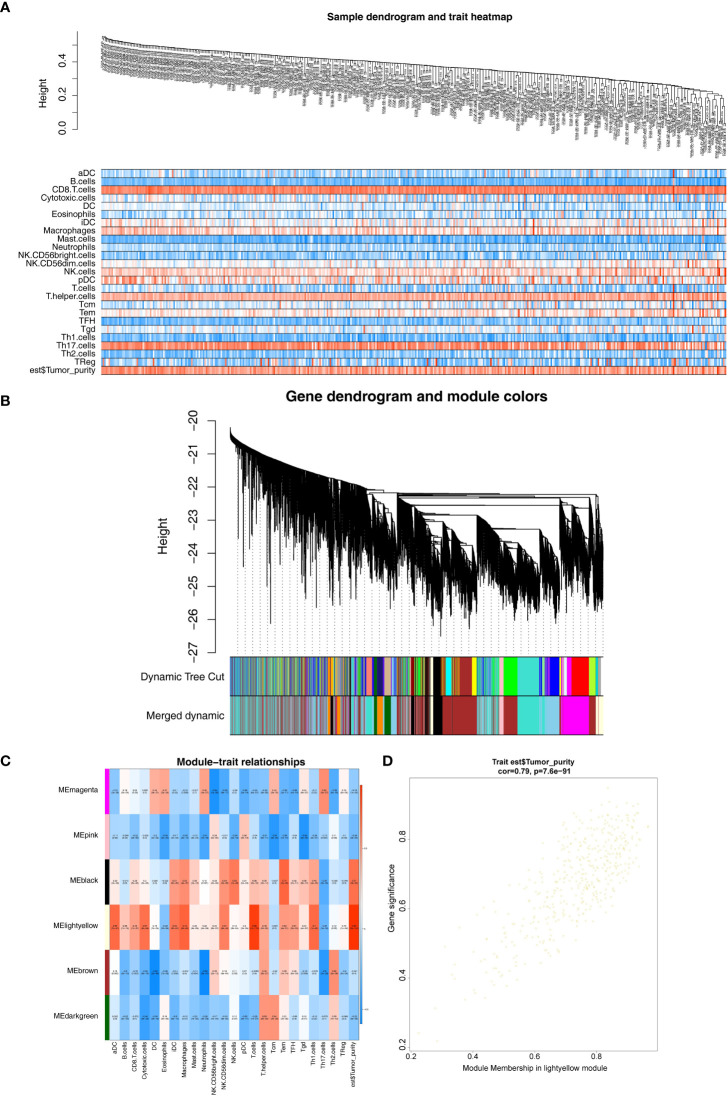
Weighted gene co-expression network analysis. **(A)** Sample and trait tree diagram. **(B)** Clustering Module Tree diagram. **(C)** Heatmap of correlations between modules and clinical traits. **(D)** A scatter plot of the gene significance (GS) versus the Module Membership (MM) in light-yellow module.

The expression profiles of 374 HCC samples from the TCGA database were selected as the basis for WGCNA. The tumor purity and immune-infiltrating cell content of all TCGA-HCC samples obtained were available in **Supplementary Figure S1**, and they would serve as the phenotype of interest for this study. The similarity of TCGA-HCC samples was detected using the goodSamplesGenes function (**Figure 2A**). A total of nine modules were obtained by WGCNA (**Figure 2B**). Subsequently, we examined the correlation of tumor purity and immune-infiltrating cells with the respective module. As indicated by the results, the light-yellow module was the highest correlated with tumor purity (cor = 0.89, *P* = 2e- 147) and also exhibited a moderate to the strong relationship with a variety of immune infiltrating cells (e.g., T cells, macrophages, Th 1 cells, aDC, cytotoxic cells, etc.) (**Figure 2C**), and the 420 genes in the light-yellow module were regarded as hub genes (**Figure 2D**).

### 3.3 Identification and enrichment analysis of the tumor purity–associated genes with differential expression

**Figure 3:**
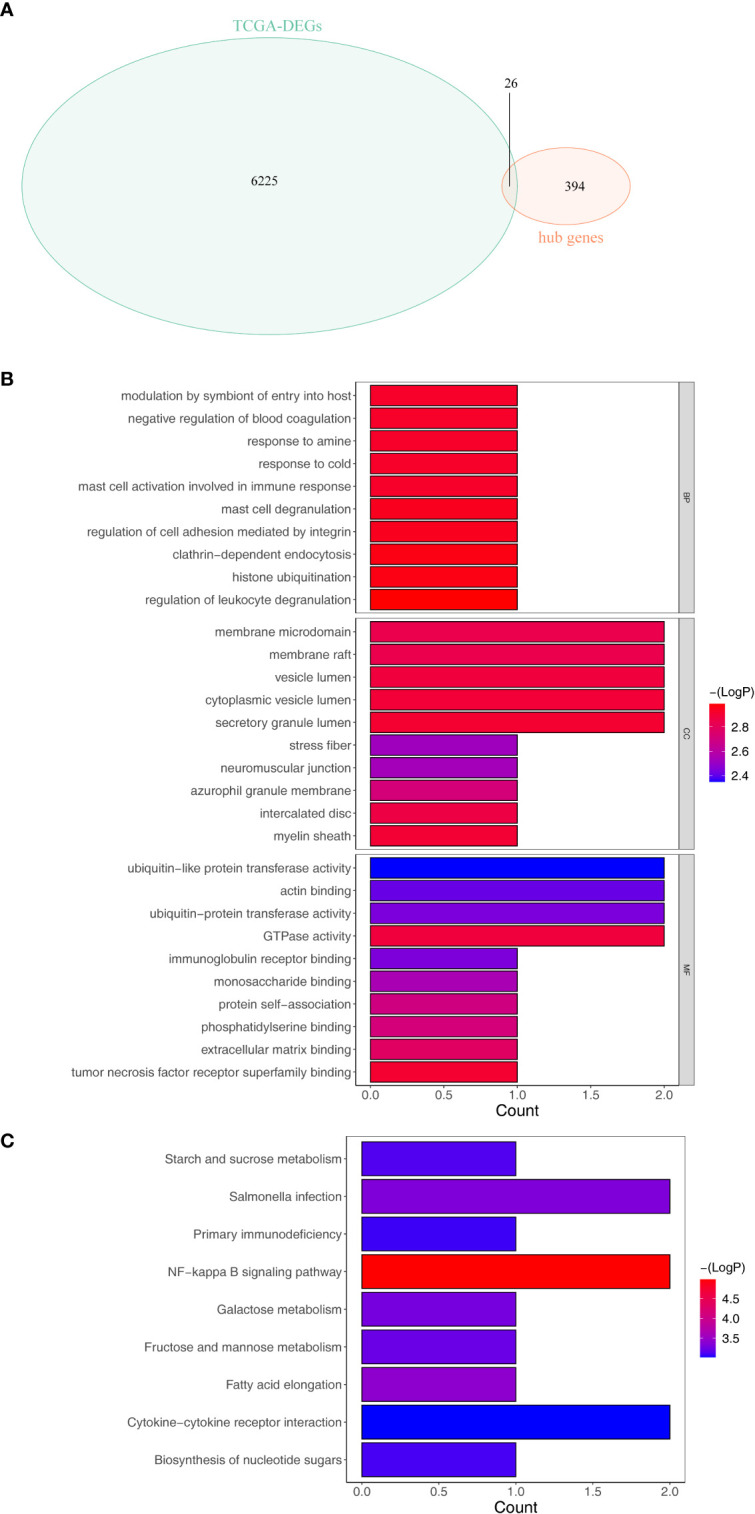
Identification and enrichment analysis of the differentially expressed tumor purity–associated genes (tumor purity–associated DEGs). **(A)** Venn diagrams of DEGs and hub genes within the light-yellow module. **(B)** GO enrichment analysis. **(C)** KEGG enrichment analysis.

Overlap analysis revealed a total of 26 common genes (**Supplementary Table S4**) were found between DEGs and hub genes as shown in **Figure 3A**, and they were defined as the tumor purity–associated DEGs. Moreover, the 26 tumor purity–associated DEGs enriched 10 GO terms and nine KEGG pathways. For instance, in the BP group, they were mainly involved in neutrophil activation, degranulation, and their mediated immunity, and granulocyte migration; in the CC category, the “tertiary granule,” “ficolin- 1-rich granule,” “ficolin-1-rich granule membrane,” and “tertiary granule membrane” were notably enriched; in the MF group, the above-described genes were closely correlated with “RAGE receptor binding” (**Figure 3B**). According to KEGG pathway enrichment analysis (**Figure 3C**), the abovementioned tumor purity–associated DEGs were involved in immune/inflammatory responses (“NF-kappa B signaling pathway,” “Primary immunodeficiency,” and “Cytokine–cytokine receptor interaction”) and metabolism (“Galactose metabolism,” “Fructose and mannose metabolism,” and “starch and sucrose metabolism”)-associated pathways, “salmonella infection,” and “biosynthesis of nucleotide sugars”; moreover, surprisingly, “fatty acid elongation” pathway also appeared notably enriched. Recently, it was reported that fatty acid extension from C16 to C18 can promote hepatic lipid accumulation and inflammation thereby promoting liver disease (21–23). The above evidence suggested that the above-described tumor purity–associated DEGs may affect the immune and inflammatory responses of patients by regulating fatty acid elongation and thus play an important role in HCC progression.

### 3.4 Identification of tumor purity–associated prognostic genes

**Figure 4:**
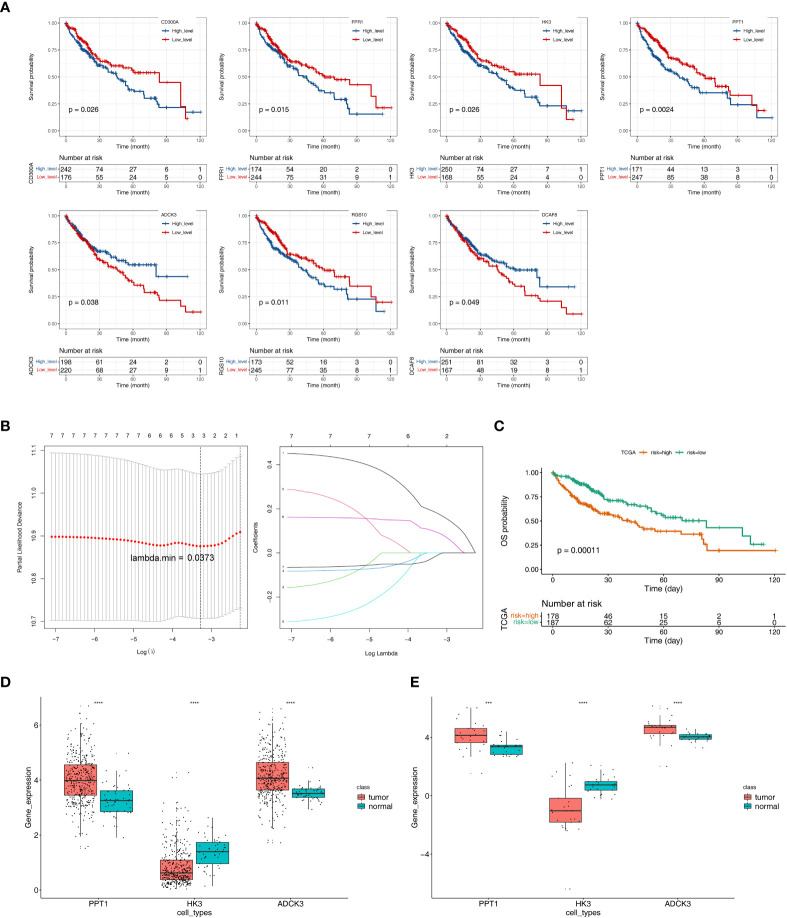
Identification of tumor purity–associated prognostic genes. **(A)** K–M survival analysis of 26 tumor purity–associated DEGs with OS in TCGA-HCC patients. **(B)** LASSO regression analysis. **(C)** Survival analysis of high- and low-risk groups. **(D)** The expression levels of three key prognostic genes in the TCGA-HCC cohort. **(E)** The expression levels of three key prognostic genes in GSE105130 dataset. ***p< 0.001, ****p< 0.0001.


We performed a K–M survival analysis designed to assess the correlation of changes in expression of 26 tumor purity–associated DEGs with OS in TCGA-HCC patients (*n* = 342). As indicated by the results, the expression of CD300A, FPR1, HK3, PPT1, and RGS10 was inversely correlated with patient survival, and high expression of the above-described genes was notably related to short survival time; patients with high ADCK3 and DCAF8 expression had notably better OS than those in the low-expression group (**Figure 4A**). The expression changes of the remaining 19 genes could not notably differentiate the clinical outcomes of HCC patients (**Supplementary Figure S3**). Subsequently, we included the seven genes mentioned above that were notably correlated with HCC prognosis to the LASSO regression analysis. Ultimately, ADCK3, HK3, and PPT1 were identified as prognostic genes based on λ min = 0.0373 (**Figure 4B**). In addition, based on Equation 2:

(2) Risk Score=(-0.01588)×expression ADCK3+0.183293×expression PPT1+0.09556×expression HK3

The optimal threshold of 5.65, and the cut point of maximally selected rank statistics = 0.73, the HCC samples were divided into high- (*n* = 178) and low-risk (*n* = 187) groups. Furthermore, the survival analysis result illustrated that the OS of low-risk group was notably higher than high-risk group (*p* = 0.00011) (**Figure 4C**).

Next, the expression levels of three key prognostic genes were compared between HCC samples and controls in the TCGA-HCC cohort and GEO150130. In both datasets, HCC tissues had lower HK3 expression and higher PPT1 and ADCK3 expressions than paired adjacent non-tumor tissues (**Figures 4D–E**). To further validate the prognostic gene expression trends experimentally, we utilized cell lines and qRT-PCR technique. As indicated by the results, in agreement with the results of the public database tissue, PPT1 and ADCK3 expression was upregulated in three hepatocellular carcinoma cell lines (Huh-1, HepG2, and SK-HeP-1) in comparison with normal hepatocytes LX-2 (**Supplementary Figure S4**). However, probably due to differences in cell lines and tissues, the expression trend of HK3 in cell lines was opposite to that in tissues (**Supplementary Figure S4**). The expression levels of three key prognostic genes were also validated in the GSE36376 dataset and achieved a consistent result (**Supplementary Figure S5A**). The AUC values of the prognostic model at 1, 2, 3, 4, and 5 years reached 0.68, 0.64, 0.60, 0.60, and 0.59, respectively (**Supplementary Figure S5B**).

Furthermore, we analyzed the correlation of the three prognostic genes exhibiting the clinical characteristics (TNM phase and phase) of TCGA-HCC samples. As indicated by the results, only PPT1 was notably correlated with the phase (*P* = 0.0033) and T-phase (*P* = 0.0014). In the phase subgroup, the expression level of PPT1 gradually increased in patients at phase I to phase III but decreased in patients at phase IV. In the T-phase subgroup, PPT1 expression increased with increasing the tumor size and depth of infiltration (**Supplementary Figure S6**).

### 3.5 Development of a nomogram

**Figure 5:**
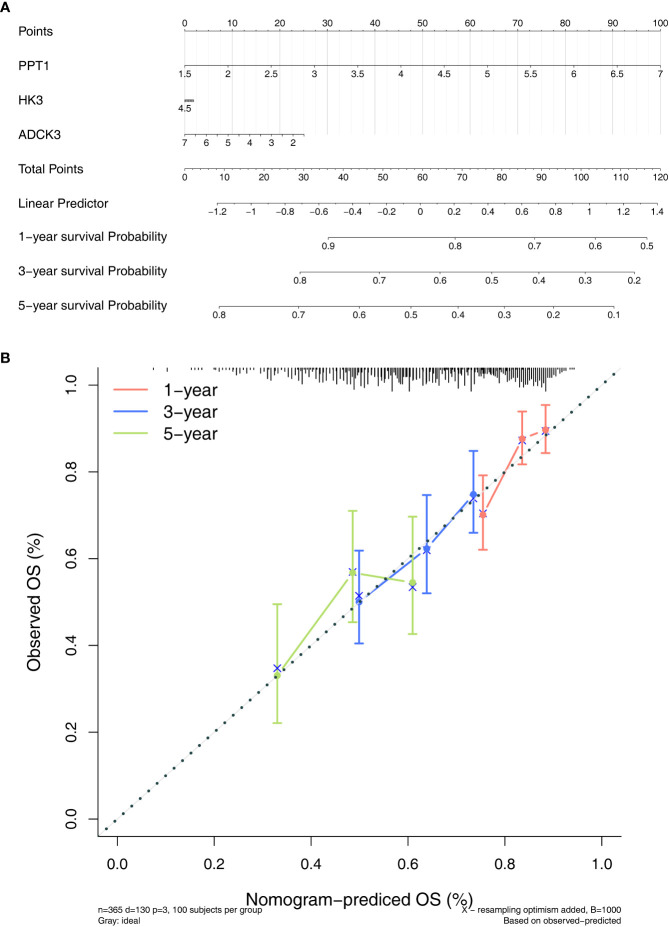
Establishment of a nomogram. **(A)** Nomogram for predicting the survival rate of patients with HCC based on three prognostic genes. **(B)** Calibration curve plotted to evaluate consistency of predicted and actual observations.

To evaluate the role played by the prognostic model, a nomogram was developed with an optimal concordance index (C-index, 0.61353) for the prediction of the survival time of HCC patients at 1, 3, and 5 years (**Figure 5A**). The calibration diagram was plotted, closer to the ideal curve, suggesting the perfect stability of the nomogram (**Figure 5B**). The above results demonstrated that the nomogram based on three prognostic genes could supply a high value for predicting the prognosis of patients with HCC.

### 3.6 Correlation of prognostic genes with immunophenotypes

**Figure 6:**
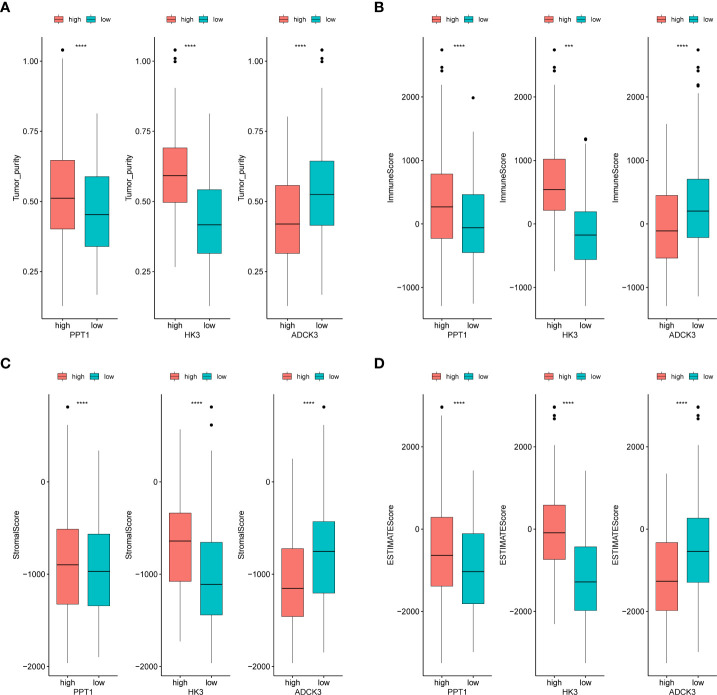
Identification of tumor purity–associated prognostic genes. **(A)** Differences in tumor purity between high- and low-expression groups of prognostic genes. **(B)** Differences in immune score between high- and low-expression groups of prognostic genes. **(C)** Differences in stromal score between high- and low-expression groups of prognostic genes. **(D)** Differences in ESTIMATE score between high- and low-expression groups of prognostic genes. ***p< 0.001, ****p< 0.0001.

We evaluated the immunophenotype of three prognostic genes. As indicated by the results, ADCK3, HK3, and PPT1 were notably correlated with tumor purity, immune score, stromal score, and ESTIMATE score (**Figure 6**). High HK3 and PPT1 expression and low ADCK3 expression resulted in high tumor purity, high immune score (**Figure 6A**), high immune score (**Figure 6B**), high stromal score (**Figure 6C**), and high ESTIMATE score (**Figure 6D**). Scatter plots of the correlations between the abovementioned prognostic genes and tumor environment scores were shown in **Supplementary Figure S7**.

### 3.7 Exploration of the potential molecular mechanisms for prognostic genes

**Figure 7:**
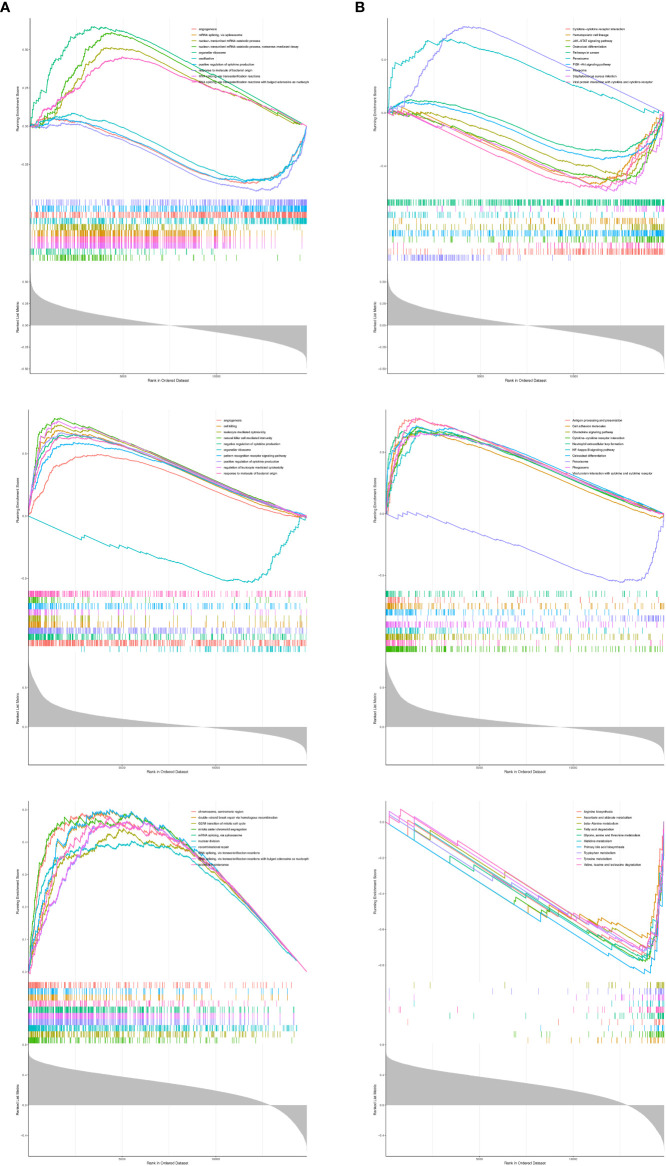
The top 10 terms of each prognostic gene in GSEA analysis. **(A)** GO analysis. **(B)** KEGG analysis.

To reveal the molecular mechanisms of prognostic genes, we performed a single-gene GSEA on three prognostic genes. ADCK3 was enriched for a total of 2,172 GO terms (1659 BP terms, 245 CC terms, and 268 MF terms; **Supplementary Table S5**, sheet1); HK3 was enriched for a total of 1,669 GO terms (1,374 BP terms, 124 CC terms, and 171 MF terms; **Supplementary Table S5**, sheet2); PPT1 was enriched with a total of 1,341 GO terms (964 BP terms, 183 GO terms, and 194 MF terms; **Supplementary Table S5**, sheet3). **Figure 7A** showed the top 10 terms of each prognostic gene in the GO Overall; all three prognostic genes were closely correlated with immune cell physiological processes (e.g., differentiation, activation, proliferation, adhesion, and related regulatory signals), immune-inflammatory response, cell cycle, vascular growth, and fatty acid production. The top 10 KEGG pathways of the three prognostic genes were displayed in **Figure 7B**, and they were notably correlated with immune-inflammatory response-associated pathways, also involved in Fatty acid degradation, Tyrosine metabolism, and Primary bile acid biosynthesis. Exhaustive KEGG enrichment results for ADCK3, HK3, and PPT1 could be reviewed in **Supplementary Table S6**.

### 3.8 Identification of differential immune cells and correlation analysis

**Figure 8:**
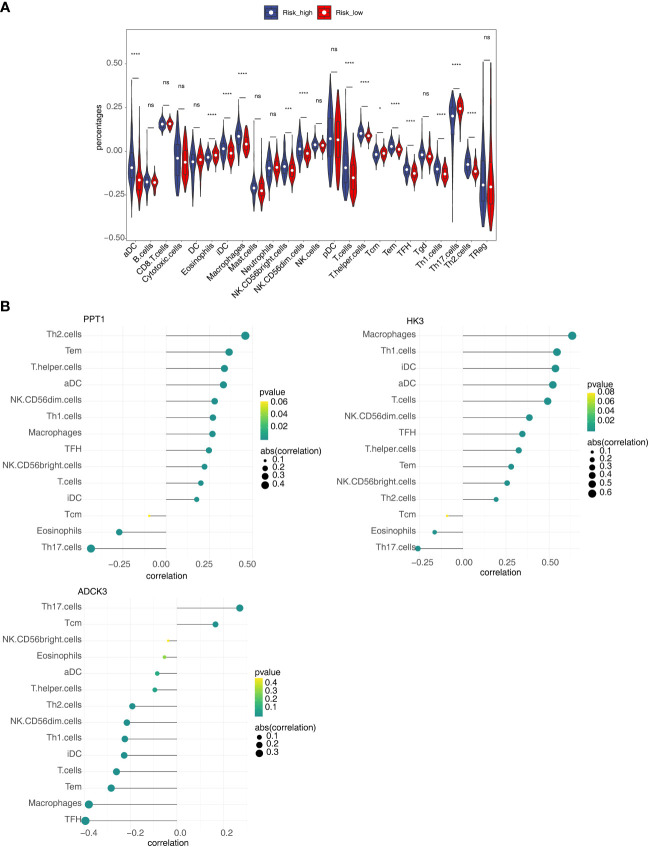
Relevance of three prognostic genes and 14 differential immune cells. **(A)** The proportion of 24 immune cells in high- and low-risk groups. **(B)** The relevance of three prognostic genes (PPT1, HK3, and ADCK3) to 14 differential immune cells. ns, not significant; *p< 0.05, ***p< 0.001, and ****p< 0.0001.

To clarify the correlation between the prognostic model and immune infiltration, we recognized the differential immune cells between high- and low-risk subgroups. **Figure 8A** revealed the proportion of 14 immune cells [eosinophils, aDC, iDC, macrophages, CD56 bright natural killer (NK) cells, CD56 dim NK cells, T cells, T-helper cells, Tcm, Tem, TFH, TH1 cells, TH2 cells, and Th17 cells] was notably different between the high- and low-risk groups. In addition, most of the differential immune cells were positively correlated with PPT1 and HK3 but negatively correlated with ADCK3 (**Figure 8B**). Thus, differential immune cells might be linked with occurrence and development of HCC.

## 4 Discussion

Hepatocellular carcinoma (HCC) is the most common primary liver cancer with poor prognosis. The incidence of HCC and HCC-associated deaths have increased over the past several decades (24). Consequently, there is an urgent need to find prognostic markers for HCC. Previous studies have shown that tumor purity is correlated with patient prognosis (11, 25). Tumor purity refers to the proportion of cancer cells in the tumor tissue. Several computational methods that can determine tumor purity have been introduced with the advance of genomics, which made the measurement of tumor purity more objective and accurate. In accordance with the ESTIMATE algorithm, tumor purity was estimated based on immune score and stromal score. Tumor immune score is an important factor affecting tumor progression and immunotherapy outcomes (26). In this study, ESTIMATE was used to calculate tumor purity of each HCC sample in TCGA-LIHC cohort. Through the ssGSEA algorithm, the immune activity of the respective sample can be accurately obtained.

In this study, we screened out three key prognostic genes; they are PPT1, HK3, and ADCK3. Palmitoyl-protein thioesterase 1 (PPT1) is an enzyme that cleaves thioester-linked palmitate from S-acylated proteins in lysosomes (27), Palmitoyl-protein thioesterase 1 (PPT1) was transported to lysosomes through the mannose-6-phosphate receptor-mediated pathway, and it participates in the lysosomal degradation (28). In addition, PPT1 is known to be widely and notably overexpressed in a variety of cancers, including breast, thyroid, and gastric cancers. In addition, higher expression levels of PPT1 in tumors are correlated with shorter overall survival for a variety of cancers. In our study, PPT1 expression was highly expressed in HCC tissues compared with normal tissue. Meanwhile, PPT1 expression in clinical samples is correlated with worse prognosis. Survival analyses for TCGA cancer patients also demonstrated that tumor expression of PPT1 was correlated with shorter overall survival in HCC (29). Meanwhile, PPT1 expression in clinical samples is correlated with worse prognosis. Therefore, we may infer that HCC patients exhibiting higher expression of PPT1 had a worse prognosis. In addition, PPT1 was notably enriched in pathways such as fat metabolism. There are studies that have indicated that, in variety of cancers, fat uptake, storage, and fat production are upregulated, which in turn promotes the rapid growth, invasion, and migration of tumors (30).The above-described results suggest that PPT1 gene may affect the occurrence and development of HCC through fat metabolism. Also, multiple substance synthesis pathways promotes the progression and malignant behaviors of cancers (31).

Hexokinase-3 (HK3), that is, a member of the hexokinase family, is involved in the first step of glucose metabolism, and its coding gene is located on the human chromosomal 5q35.2 segment. The inactivation of HK3 notably affects the activation of cancer cells glycolysis, and then activates the downstream signaling pathways, such as apoptosis and endoplasmic reticulum stress. In cancer cells, which plays a vital role in the progression and development of cancers (32). Also, in numerous studies, HK3 has been identified as a potential marker for regulating the tumor metabolic microenvironment and malignant progression, with predictive efficacy for tumor progression and prognosis. In addition, low expression of HK3 were usually malignant entities and were shown to be obvious genomic aberrations of driver oncogenespoor (33). In this study, from enrichment analysis of GSEA, the HK3 gene was notably enriched in cancer pathways, cellular communication factor, PI3K-Akt Pathway, and virus response. Cancer pathway takes on critical significance to cancer progression and cancer-associated genes’ expression, regulating tumor progression and prognosis (34). Furthermore, the validation of expression level in the GEO external dataset confirmed that the HK3 gene was upregulated in HCC compared with normal groups, consistent with previous studies, suggesting higher expression of HK3 in HCC patients with poor prognosis.

ADCK3 has been confirmed as a mitochondrial protein homologous to the yeast COQ8 and the bacterial UbiB proteins, required for CoQ biosynthesis. Amount of experiments strongly suggested that ADCK3 is also involved in CoQ10 biosynthesis (35). CoQ10 may play a certain role in regulating apoptosis (36, 37). CoQ10, an energy transfer molecule, occurs in high levels in the liver. As reported by some research, a possible inverse correlation exists between blood CoQ 10 levels and cancer (38). In the above research, similar to the HK3 gene, the ADCK3 gene also enriched in cancer pathways, cellular communication factor, PI3K-Akt Pathway and virus response. The PI3K-Akt Pathway is closely related to the malignant behavior of tumor cells, and it manifested its compelling influence on multiple cellular process in different cancers, which are closely related with tumorigenesis, proliferation, growth, apoptosis, invasion, metastasis, epithelial–mesenchymal transition, stem-like phenotype, immune microenvironment and drug resistance of cancer cells (39). Moreover, the PI3K/Akt pathway plays an important role in tumor formation and metastasis. Various analysis identified, PI3K/Akt mutation status can be used as a novel predictor of cancer patients (40). Our research in GEO external validation dataset also identified ADCK3 showed high-expression level in cancer tissue in comparison with normal tissue. Thus, we can speculate that better prognosis of HCC patients correlated with high ADCK3 expression.

In summary, our study identified the abovementioned TME-associated prognostic markers in HCC. PPT1 and HK3 were overexpressed in tumor and its high expression was correlated with poor prognosis, while high ADCK3 expression was correlated with better survival. However, the prognostic value of the three genes warrants further validation by more clinical data. Importantly, the HK3 and ADCK3 genes were not only highly expressed in HCC but also correlated with PI3K/Akt Pathway and cancer pathways. It holds a great potential as a candidate for targeted immunotherapy of HCC.

## 5 Conclusions

In conclusion, this study identified three novel predictive biomarkers (ADCK3, HK3, and PPT1) correlated with tumor purity for HCC by bioinformatics methods. In addition, the nomogram was established based on three biomarkers to predict the survival time of HCC patients at 1, 3, and 5 years. The current study might contribute to the prognostic workup of HCC and played a complementary role in determining tumor purity phenotypes.

## Data availability statement

The datasets presented in this study can be found in online repositories. The names of the repository/repositories and accession number(s) can be found in the article/**Supplementary Material**.

## Author contributions

YZ and RX contributed equally to this work. YZ and LW conducted data collection, procession and analysis. RX and YZ, XX conducted result summaries, visualizations, and validation. All the authors contributed to the manuscript’ s development.

## Supplementary material

Supplementary Figure 1 Distribution of immune infiltrating cells in all samples. The horizontal axis represents the respective sample, the vertical axis represents the size of the immune cell score, and different colors represent different immune cells.

Supplementary Figure 2 Weighted gene co-expression network analysis. **(A)** Screening of scale-free soft thresholds. The horizontal axis of the above graphs all represent the weight parameter power value, and the vertical axis of the left figure represents the square of the correlation coefficient of log(k) and log[p(k)] in the relevant network, that is, signedR2, the vertical axis of the right graph represents the mean of the adjacency functions of all genes in the relevant gene module. **(B)** Clustering of module Eigen genes.

Supplementary Figure 3 K–M survival analysis of the 19 differentially expressed tumor purity–associated genes (tumor purity–associated DEGs).

Supplementary Figure 4 PPT1 and ADCK3 expression was upregulated in three hepatocellular carcinoma cell lines (Huh-1, HepG2, and SK-HeP-1) in comparison with normal hepatocytes LX-2.

Supplementary Figure 5 Validation of the expression levels of prognostic genes and the prognostic model. **(A)** The expression levels of prognostic genes in the GSE36376 dataset. *****p*< 0.0001. **(B)** The ROC curve of the prognostic model.

Supplementary Figure 6 Difference analysis of three model genes in different clinical groups (TNM phase and phase) of TCGA-HCC samples.

Supplementary Figure 7 The correlations between prognostic genes and tumor environment scores, **(A)** tumor purity, **(B)** immune score, **(C)** stromal score, **(D)** ESTIMATE score.

Supplementary Table 1 The clinical and survival information of samples in TCGA dataset.

Supplementary Table 2 Tumor purity and immune infiltrating cell abundance in 374 HCC samples.

Supplementary Table 3 Differentially expressed genes (DEGs) between HCC samples and normal samples.

Supplementary Table 4 The tumor purity–associated DEGs in HCC.

Supplementary Table 5 The GO enrichment results of ADCK3, HK3, and PPT1.

Supplementary Table 6 The KEGG enrichment results of ADCK3, HK3, and PPT1.
