# Supplementary figures and images for: Tumor purity–associated genes influence hepatocellular carcinoma prognosis and tumor microenvironment
Source: Front Oncol. 2023 Jun 26;13:1197898. doi: 10.3389/fonc.2023.1197898 (PMC10330704; doi:10.3389/fonc.2023.1197898)

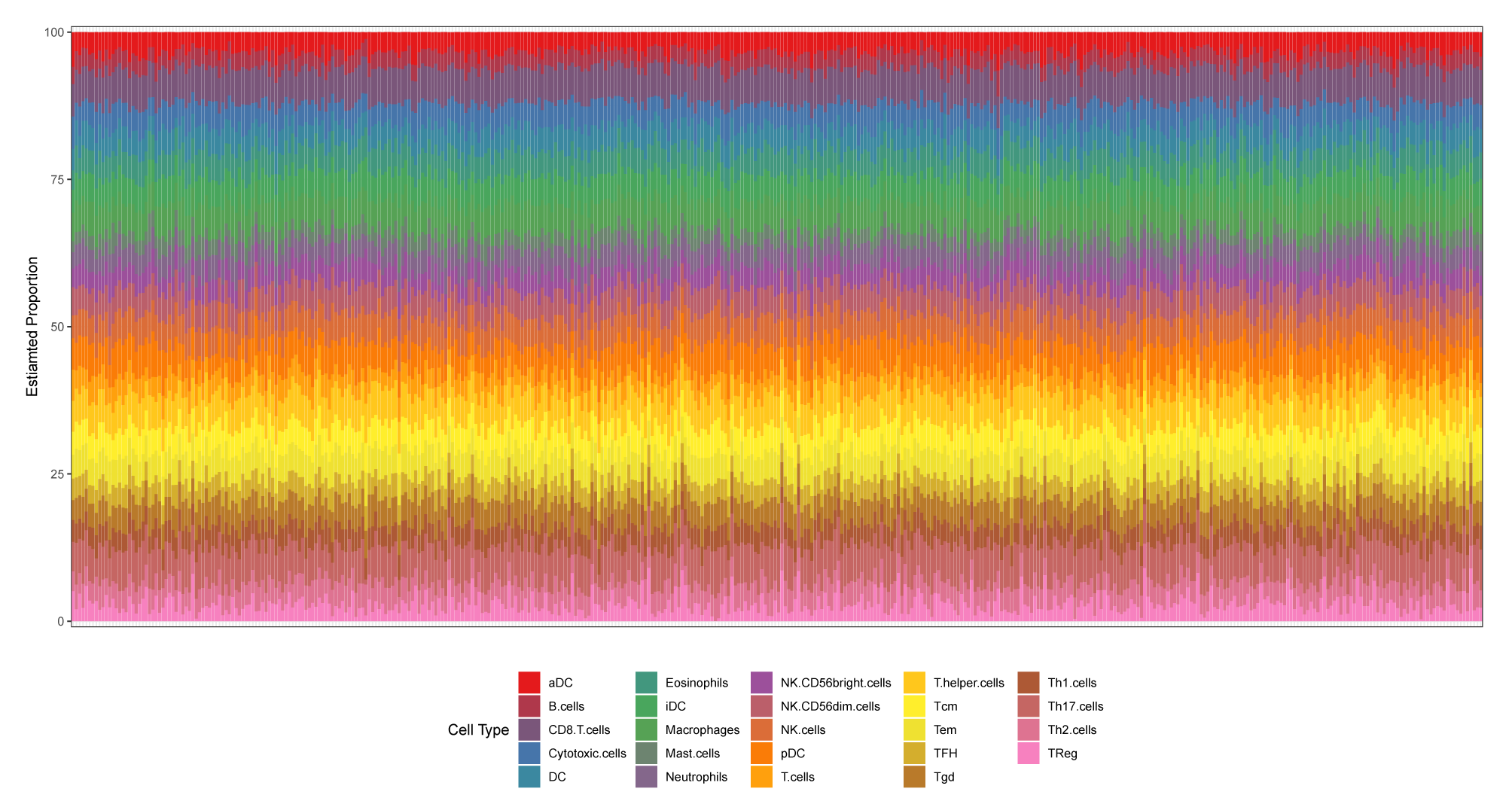

Supplement: Supplementary Figure 1 — Distribution of immune infiltrating cells in all samples. The horizontal axis represents the respective sample, the vertical axis represents the size of the immune cell score, and different colors represent different immune cells. [file Image_1.tif]

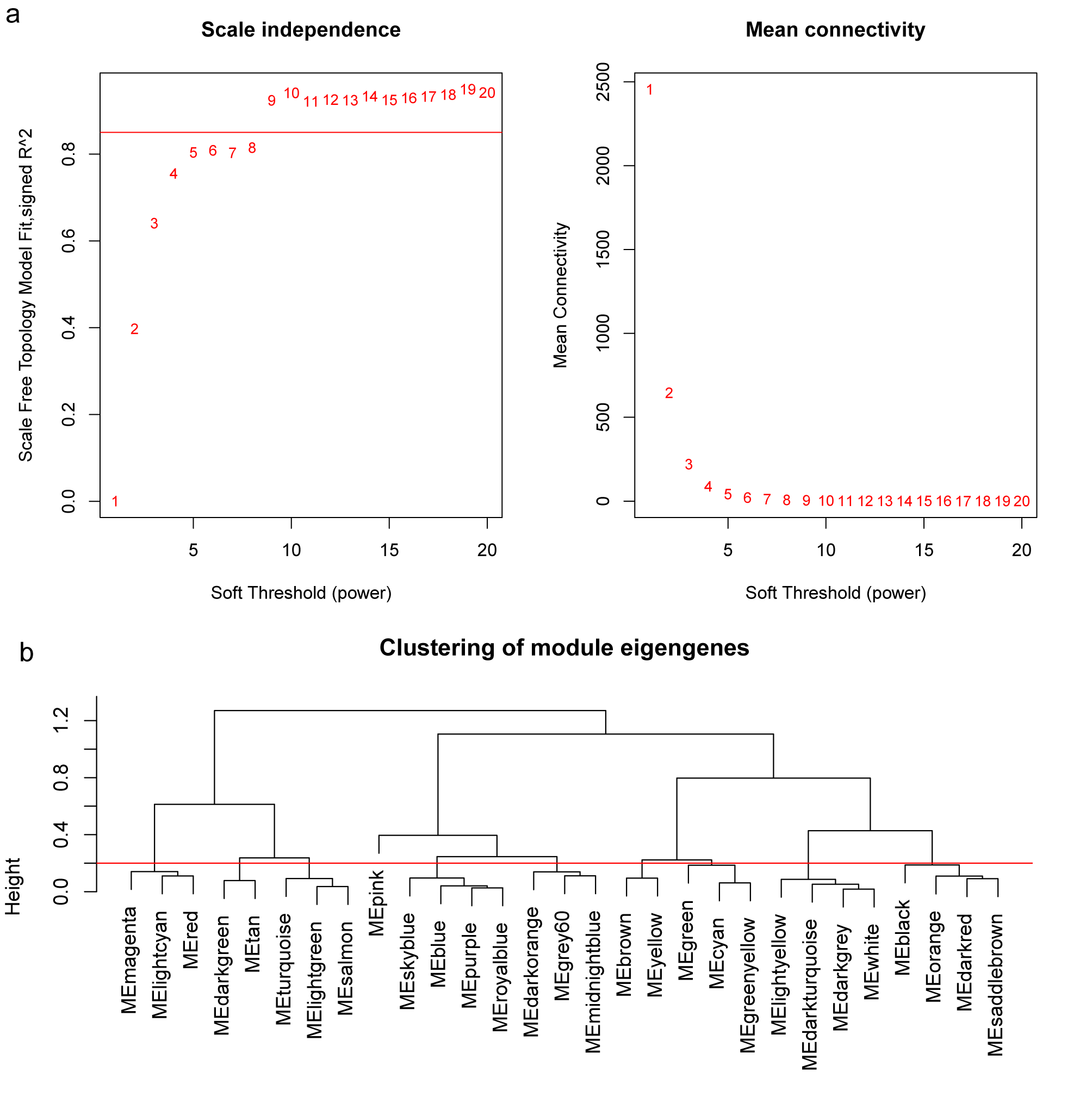

Supplement: Supplementary Figure 2 — Weighted gene co-expression network analysis. (A) Screening of scale-free soft thresholds. The horizontal axis of the above graphs all represent the weight parameter power value, and the vertical axis of the left figure represents the square of the correlation coefficient of log(k) and log[p(k)] in the relevant network, that is, signedR2, the vertical axis of the right graph represents the mean of the adjacency functions of all genes in the relevant gene module. (B) Clustering of module Eigen genes. [file Image_2.tif]

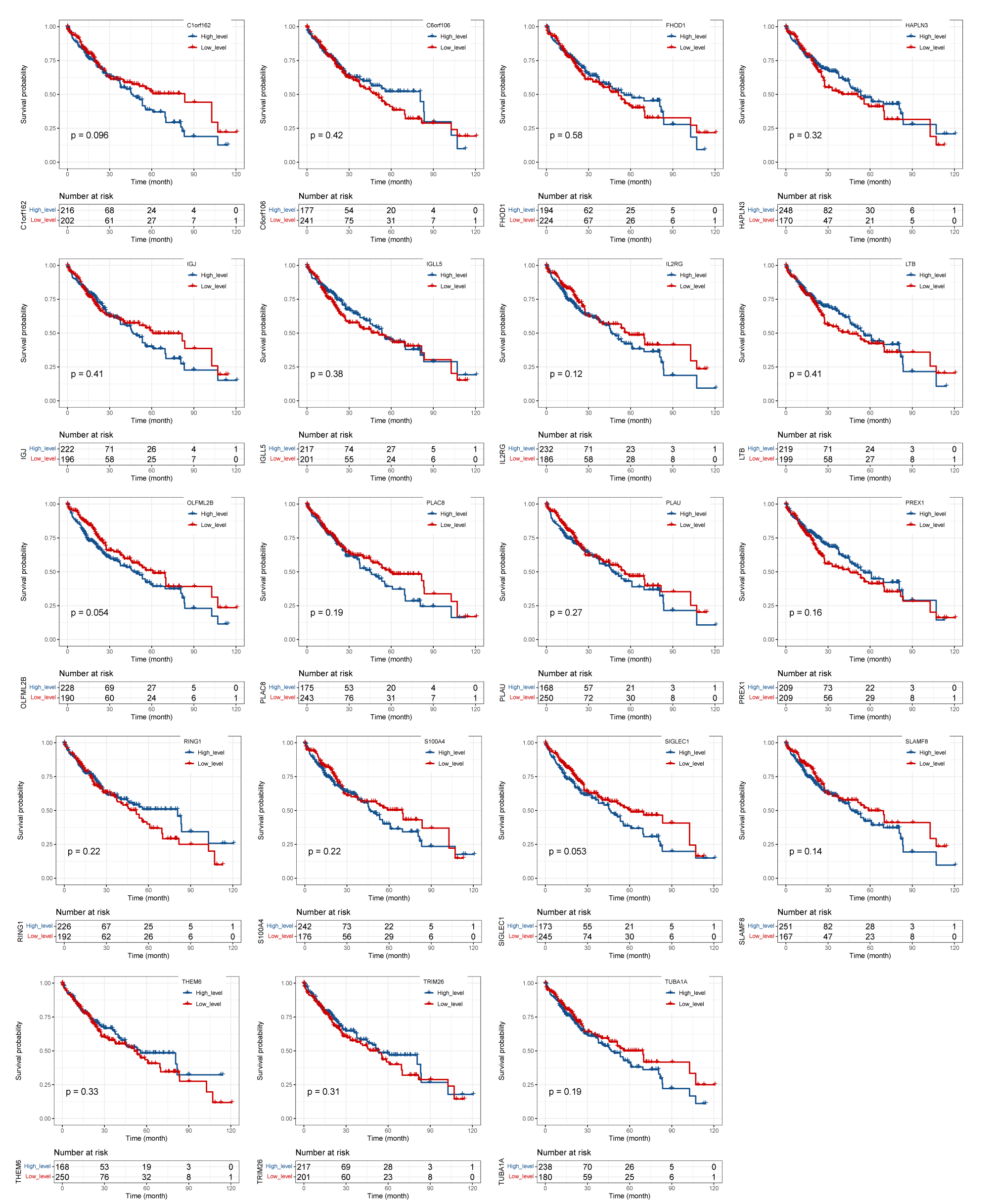

Supplement: Supplementary Figure 3 — K–M survival analysis of the 19 differentially expressed tumor purity–associated genes (tumor purity–associated DEGs). [file Image_3.tif]

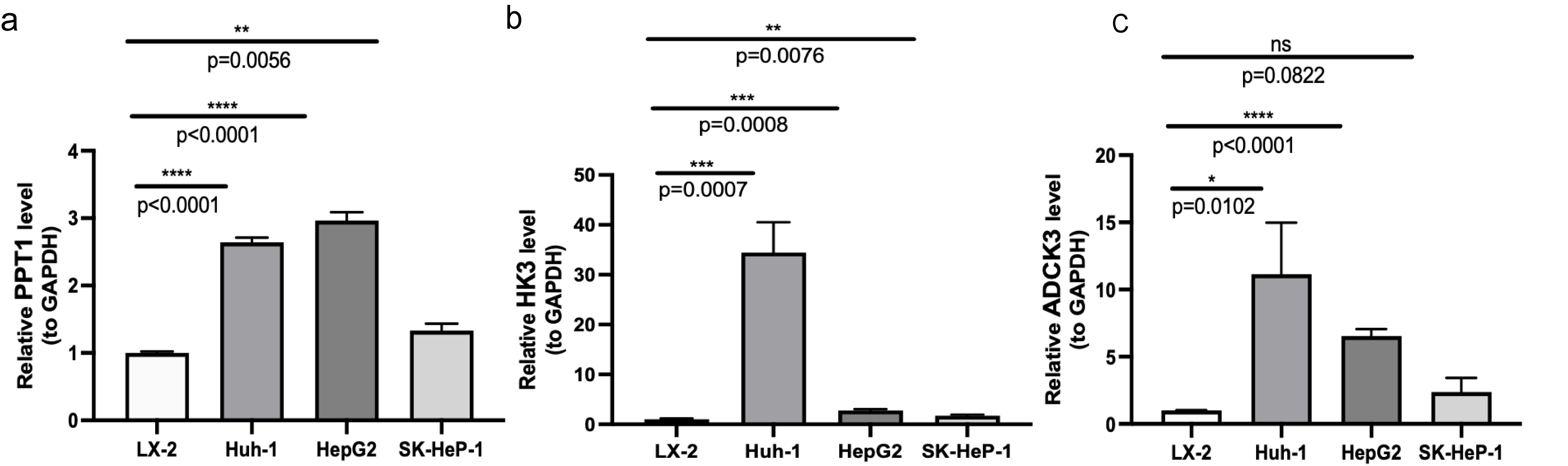

Supplement: Supplementary Figure 4 — PPT1 and ADCK3 expression was upregulated in three hepatocellular carcinoma cell lines (Huh-1, HepG2, and SK-HeP-1) in comparison with normal hepatocytes LX-2. [file Image_4.tif]

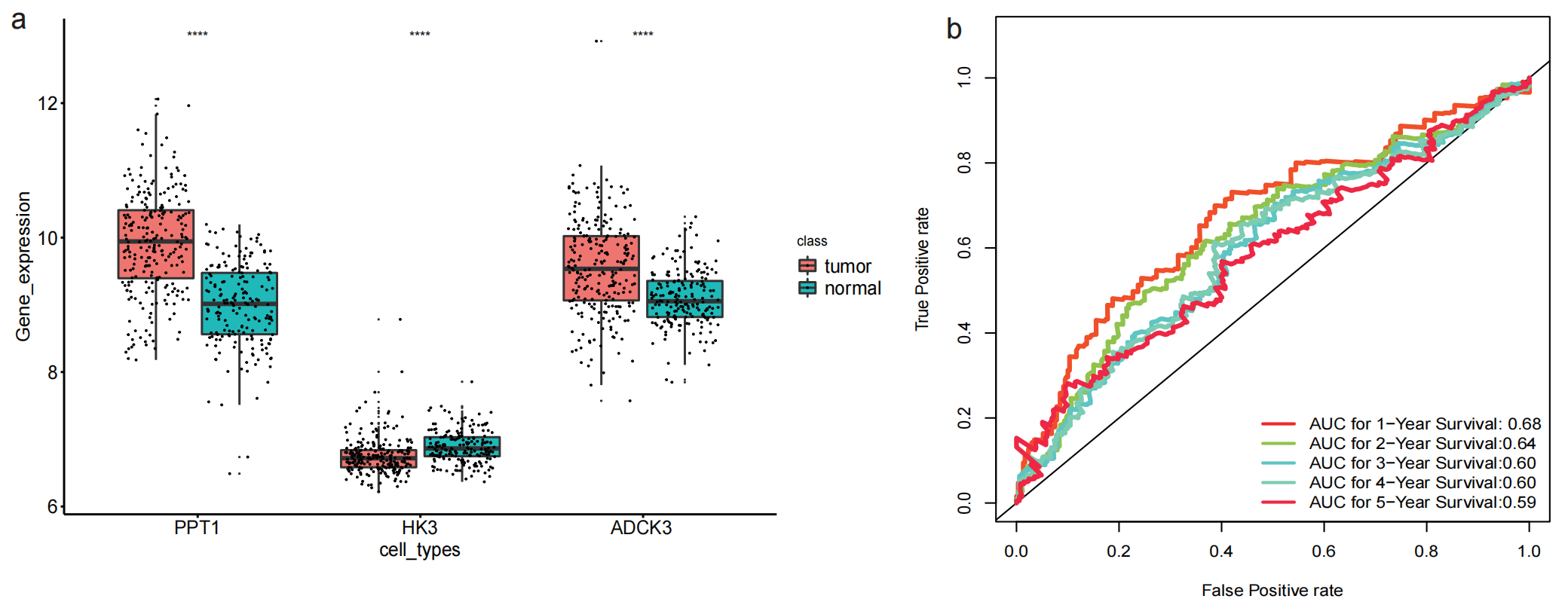

Supplement: Supplementary Figure 5 — Validation of the expression levels of prognostic genes and the prognostic model. (A) The expression levels of prognostic genes in the GSE36376 dataset. ****p< 0.0001. (B) The ROC curve of the prognostic model. [file Image_5.tif]

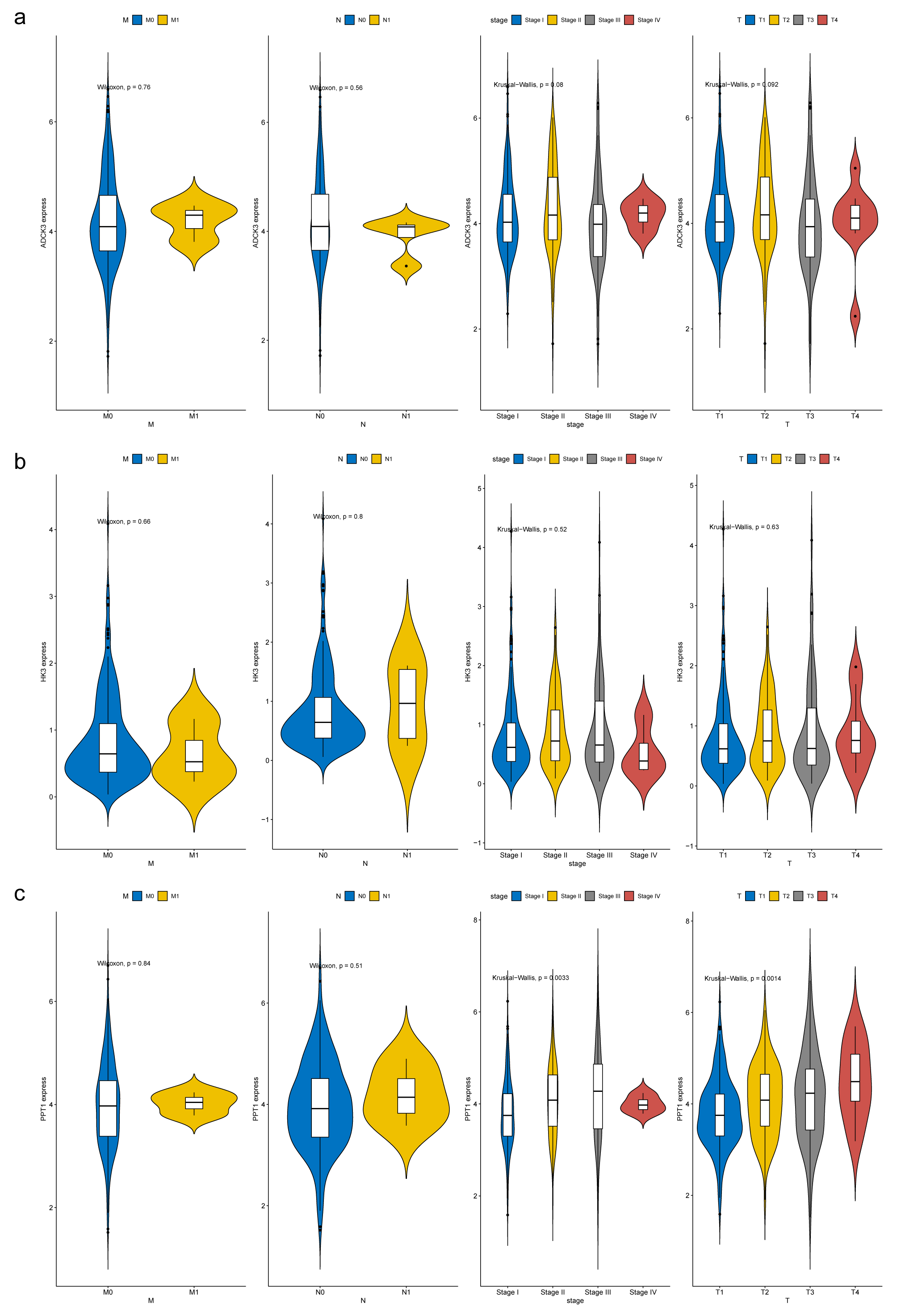

Supplement: Supplementary Figure 6 — Difference analysis of three model genes in different clinical groups (TNM phase and phase) of TCGA-HCC samples. [file Image_6.tif]

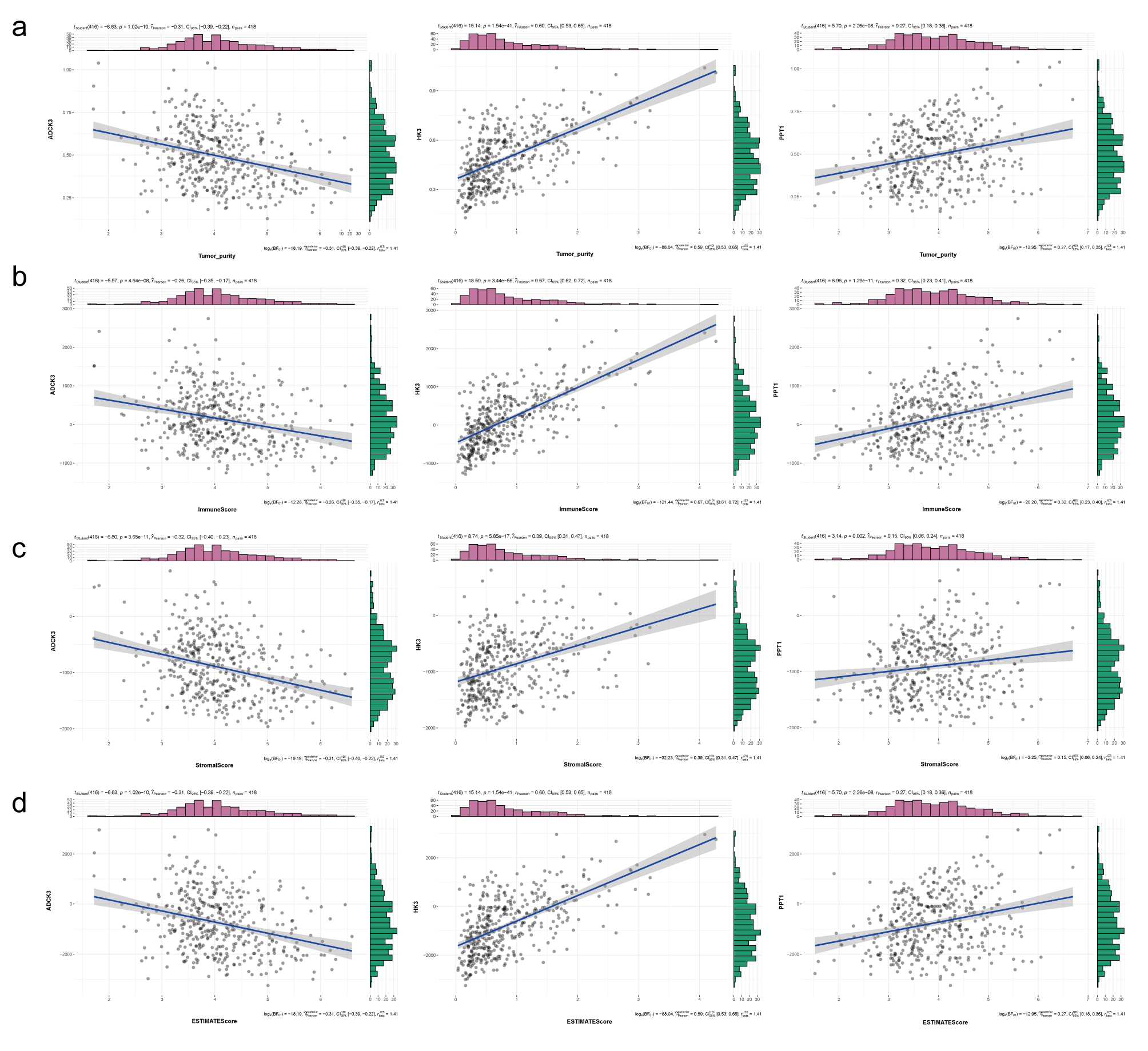

Supplement: Supplementary Figure 7 — The correlations between prognostic genes and tumor environment scores, (A) tumor purity, (B) immune score, (C) stromal score, (D) ESTIMATE score. [file Image_7.tif]
